# Everybody's Column

**Published:** 1892-02-06

**Authors:** 


### EVERYBODY'S COLUMN.

Even toy balloons have their dangers. A coloured child in the States swallowed one some time ago to its great discomfort. The result of the unfortunate accident was a severe choking and paroxysms resembling those of an epileptic. Luckily a surgeon was procured in time, and with much difficulty saved the child's life.

According to the *Journal de Médecine de Paris*, the fumes of bromhydrate of ammonium not only exert a favourable action in some forms of bronchitis, but have a most beneficial effect in cases of asthma. It is stated that after a few inhalations of fumes obtained from bromhydrate of ammonium of a specific gravity of 1·7 the difficulty in breathing disappears, and that in some cases the adoption of this form of inhalation, when used at the time of a threatened attack of asthma, has completely prevented the attack. The drug, therefore, bids fair to be a great boon to the many people who suffer from this distressing complaint.

One of the latest antidotes to the poison of the viper's bite advocated is chromic acid. This remedy is stated to have been tried with the fullest success by Dr. Kaufmann, and when introduced in proper quantities into the spot where the poisonous puncture has been made it is said to have no caustic action upon the tissues.

It is curious how articles which at first are despised as useless become not only valuable but almost indispensable commercially. This is the case with cotton seed in America. For years it was burnt as useless refuse, but only by degrees have its various uses been recognised. Of these the latest discovered is the value of the stalk, which was held unserviceable for man or beast, and which is now converted into a fibre from which paper is made. Thus by degrees the whole plant has been found to have its different uses. There are some people, perhaps, who would prefer that the oil were not quite so popular with the olive producers of Europe.

